# A Comparative Transcriptome and Proteome Analysis of the Molecular Mechanism Underlying Anterior to Dorsal Eye Rotation in the Celestial-Eye Goldfish (*Carassius auratus*)

**DOI:** 10.3390/ijms26020466

**Published:** 2025-01-08

**Authors:** Rongni Li, Yansheng Sun

**Affiliations:** Fisheries Science Institute, Beijing Academy of Agriculture and Forestry Sciences, Beijing 100068, China; vicsun69@163.com

**Keywords:** celestial-eye goldfish, antero-dorsal eye rotation, transcriptome–proteome integration, retinal degeneration, *lrp2* gene, lipid metabolic abnormalities, arginine biosynthesis, energy metabolism downregulated

## Abstract

Goldfish (*Carassius auratus*), subjected to millennia of artificial selection and breeding, have diversified into numerous ornamental varieties, such as the celestial-eye (CE) goldfish, noted for its unique dorsal eye rotation. Previous studies have primarily focused on anatomical modifications in CE goldfish eyes, yet the molecular underpinnings of their distinctive eye orientation remain poorly understood. This study employed high-throughput transcriptome and proteome sequencing on 110-day-old full-sibling CE goldfish, which displayed either anterior or upward eye rotations. Verification of these findings was conducted using quantitative PCR (qPCR) for transcriptomic data and parallel reaction monitoring (PRM) for proteomic analysis. Our research identified 73,685 genes and 7717 proteins, pinpointing 8 common differentially expressed genes (DEGs) and proteins (DEPs) implicated in cytoskeleton remodeling, cell adhesion, apoptosis, and optic nerve regeneration. Enrichment analyses further delineated pathways associated with apoptosis, necroptosis, and cell adhesion molecules. The results indicated a significant role for genes involved in cytoskeletal dynamics, nervous system function, and apoptotic processes in the dorsal eye rotation of CE goldfish. Analyses of abnormalities in ocular membrane structures, along with disturbances in lipid and protein synthesis metabolism and energy metabolism during developmental stages, provided compelling evidence for the potential use of CE goldfish as a model organism in studying human eye-related disorders. This investigation provided the first comprehensive transcriptomic and proteomic overview of eye rotation in CE goldfish, offering insights crucial for the genetic breeding of new ornamental fish varieties.

## 1. Introduction

The celestial-eye (CE) goldfish (*Carassius auratus*), known as “Wang Tian Yan” in Chinese, is one of the most revered strains of traditional goldfish, distinguished by its unique upward-facing eyes. The striking feature of this strain is its gaze, which appears to follow observers from any angle, symbolizing the ancient Chinese notion of reverence towards the emperor. During the Qing Dynasty, the CE goldfish was particularly prized in the imperial court [1], endowing it with both cultural and aesthetic significance.

Previous research on the CE goldfish primarily focused on the growth and development of its ocular structures, particularly the eyeballs and eye tissues. Some studies on the developmental process of CEs showed that its retina undergoes progressive degeneration over time, leading to restricted vision and reduced light sensitivity—similar to myopia in humans [2]. Early research, particularly from Japanese scholars, reported histological observations of the CE retina, which revealed an abnormally thin structure with a reduced number of photoreceptor cells due to macrophage infiltration. The morphology of the retina exhibited high variability both within different regions of the retina and between individual fish, and retinal degeneration progresses very slowly in CEs [3]. Subsequent studies mapped the stages of eye morphology changes, describing a transition from a flat to a rotated, antero-dorsally positioned eye, along with corresponding structural alterations in the retina [4]. Freeze-sectioning studies indicated that the axial length of the vitreous chamber in CEs accounts for more than 70% of the total axial length, while Lowry assays demonstrated that the enlargement of the eye resulted from an increase in vitreous volume, which diluted the existing vitreous material. Furthermore, the CE’s thin and fragile sclera suggested that ocular enlargement might be due to stretching of the cornea and sclera [5].

However, the molecular mechanisms underlying the spontaneous antero-dorsal rotation of the eyeball in CEs remain poorly understood. Goldfish, known for their high adaptability to diverse environments and low maintenance requirements [6], offers a promising model for studying retinal degeneration and vision impairments. The unique anatomical features of the CE goldfish make it an ideal subject for research, providing insights into the genetic and molecular pathways involved in retinal degeneration and eye abnormalities [7].

In this study, we aim to employ high-throughput sequencing technologies to perform transcriptomic and proteomic analyses of CE goldfish. Our goal is to identify candidate genes and pathways that may contribute to the eye’s rotation from anterior to dorsal. By investigating the molecular mechanisms behind the development of CE traits, we hope to not only advance our understanding of eye-related diseases but also to provide valuable genetic insights that could be applied to the breeding of ornamental fish strains in aquaculture as well as inform the prevention and treatment of ocular disorders.

## 2. Results

### 2.1. Phenotypic Observation of Eye Rotation Development in CE

We observed the phenotypic development of eye rotation in 84 juvenile full-sibling CE individuals. The following developmental milestones were noted:

At 20 days post-hatch (dph), all fish exhibited slight lateral extrusion of the eyes.

By 60 dph, the eyes of all fish had developed a telescopic-eye appearance, extruding further.

At 70 dph, approximately 12% of the fish showed the initiation of an anterior rotation of the eyes.

At 100 dph, around 40% of the individuals had fully rotated their eyes dorsally.

At 110 dph, about half of the fish demonstrated upward-facing eye rotation, with 49% having rotated anteriorly and 1% still showing no rotation.

By 280 dph, all fish had completed the upward-facing eye rotation, achieving a celestial-eye position.

To investigate the molecular changes associated with the eye rotation process, we randomly selected three goldfish with forward-facing eyes (C group) and three with upward-facing eyes (T group) at 110 days dph for subsequent molecular analysis. The reason for selecting 110 dph CE goldfish for this study was that at this stage, half of the fish had eyes that had rotated forward (T group), while the other half had eyes that had rotated upwards, forming the successfully developed CE phenotype (C group). These two eye orientations represent typical stages of eye development, with the forward-facing eyes corresponding to the mid-development stage and the upward-facing eyes corresponding to the later stage. In the T group, the fish’s eye development had rotated forward but had not yet completed the upward rotation, placing them at the intermediate developmental stage. In contrast, the C group fish had successfully undergone upward eye rotation, resulting in the characteristic “Wangtian eye“ phenotype. Therefore, selecting the T and C groups for subsequent molecular research would help to identify the molecular mechanisms underlying successful upward eye rotation and shed light on the developmental processes involved.

### 2.2. Analysis ofTranscriptome Data ofT and C Groups in CE

#### 2.2.1. Basic Transcriptome Data Analysis of Eyes with Middleand Late Rotation in CE

To identify gene expression changes during the eye rotation process in CEs, we performed transcriptomic profiling to measure mRNA abundances. The sequencing generated a total of 39.80 GB of raw data, which was processed to yield 38.52 GB of clean data after filtering. The clean reads represented over 96% of the total data, and the clean Q30 base rate exceeded 93%. The cleaned data were deposited in the China National Center for Bioinformation database (GSA: CRA020175).

The alignment of the sequencing reads to the *C. auratus* genome (https://www.ncbi.nlm.nih.gov/assembly/GCA_003368295.1) yielded an alignment rate exceeding 90%, with more than 85% of the aligned sequences localized within exonic regions (Table 1).

**Figure 1:**
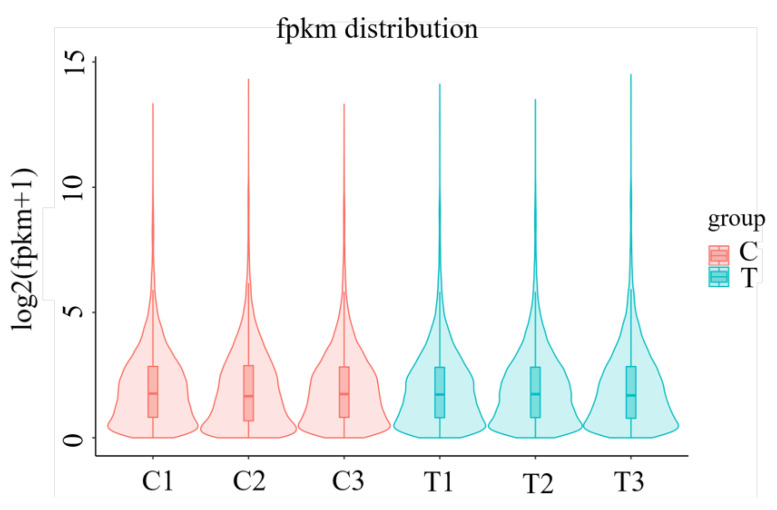
FPKM values’ violin plot.

The FPKM values’ violin plot (Figure 1) demonstrated a symmetrical distribution, indicating that gene expression followed a normal distribution. The consistency of the median values further confirmed the reliability and quality of the data, supporting its suitability for subsequent differential gene expression analysis.

#### 2.2.2. DEGs, GO, and KEGG Analysis ofTwoStagesof Eye Development in CE

**Figure 2:**
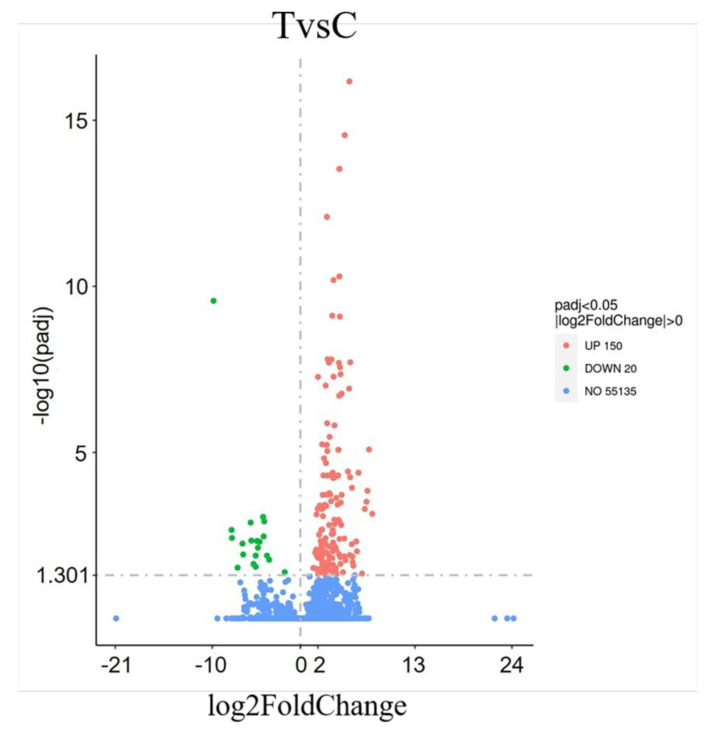
A volcano map of DEGs identified with the eye rotating forward and upward in CEs. Note: The x-axis represents log2(Fold Change), and the y-axis indicates −log10(*p*adj value); −log10*p*adj = 1.301 and *p*adj = 0.05 are considered significant differences. The vertical dashed line in the figure represents log2(Fold Change) threshold, and the horizontal dashed line represents the *p*-value = 0.05 threshold. Red plots indicate upregulated genes, and green indicate downregulated genes when comparing T with C groups.

A total of 170 DEGs were identified through a comparison between the T and C groups, with 150 genes upregulated in the T group and 20 genes downregulated (Figure 2, Table S2).

**Figure 3:**
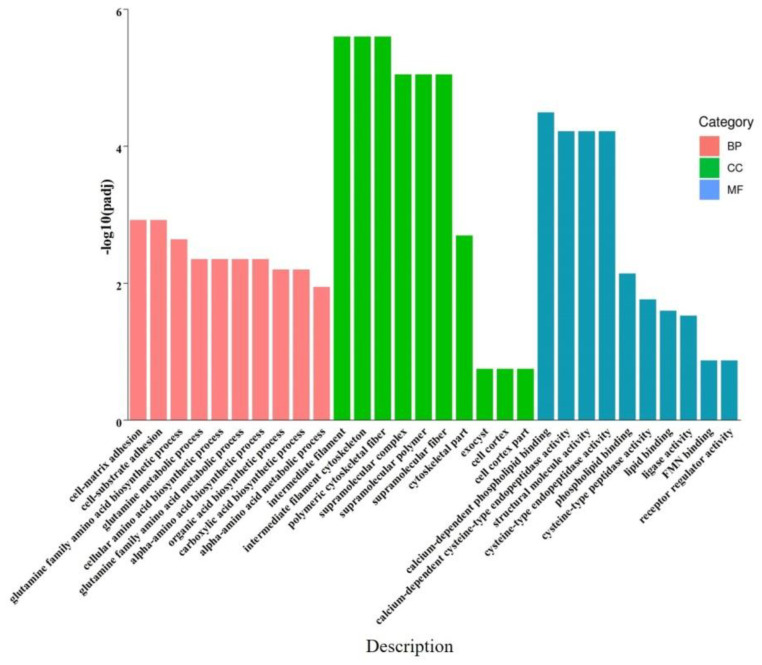
The GO analysis bar graph (T vs. C). Note: The figure displays the top 10 GO terms with the smallest *p*adj-value, which are the most significantly enriched terms, selected from each GO category. The x-axis represents the GO enrichment term descriptions, and the y-axis represents the −log10(*p*adj-value) for each term’s enrichment. The colors red, green, and blue represent the biological process (BP), cellular component (CC), and molecular function (MF), respectively.

The GO functional enrichment analysis revealed that the DEGs were significantly associated with 13 biological processes (BP), eight molecular functions (MF), and seven cellular components (CC), with a primary focus on structural molecule activity, intermediate filaments, cytoskeletal components, cell adhesion, and supramolecular complexes. The top 30 GO entries were selected for further visualization in a bar graph (Figure 3, Table S2).

**Figure 4:**
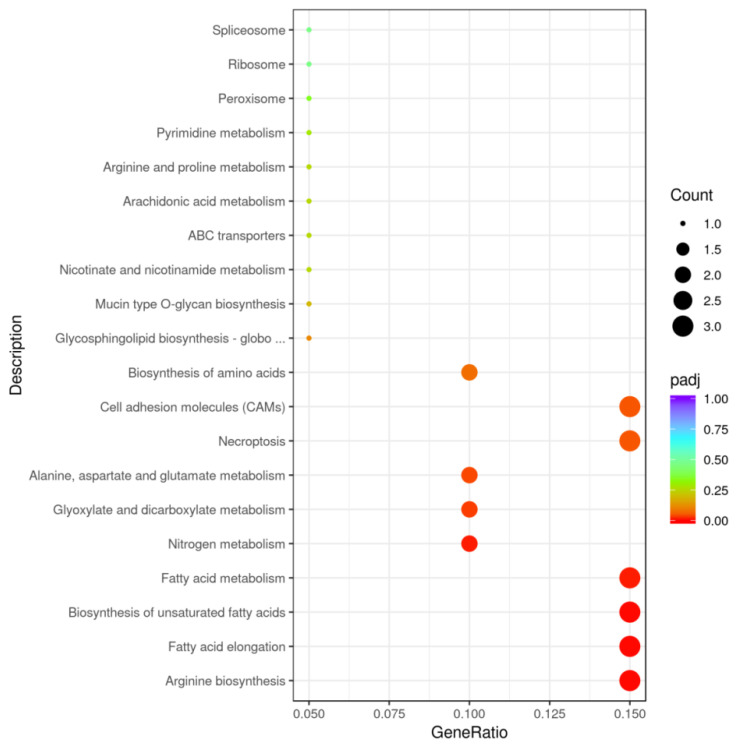
The KEGG pathway enrichment scatter plot of DEGs (T vs. C). Note: In total, 26 pathways comprised the union of the top 20 enriched pathways from T vs. C. The x-axis represents the enrichment generationof each pathway, and the y-axis shows the enrichment pathway. The color and sizesof dots indicate the *p*adj value and the number of DEGs assigned to the corresponding pathway, respectively. *p*adj < 0.05 is considered significantly enriched.

Additionally, the KEGG pathway analysis identified nine significant pathways, including those related to cell adhesion molecules, necroptosis, arginine biosynthesis, fatty acid elongation, and metabolic pathways for unsaturated fatty acids and fatty acids (Figure 4, Table S2). The nine significant pathways included a number of ten genes, whichwere *LOC113115398*, *LOC113054720*, *LOC113112742*, *LOC113065066*, *LOC113110194*, *LOC11305820*, *LOC113078755*, *LOC113038790*, *LOC113063734*, and *LOC113114746* (Table S2).

Notably, all DEGs enriched in both GO and KEGG pathways were expressed at significantly higher levels in the T group compared to the C group (Table S2), with the majority of enrichments related to cytoskeletal structure, cell adhesion, and fatty acid and amino acid metabolism as well as supramolecular complexes.

#### 2.2.3. Verification of RNA-Seq

**Figure 5:**
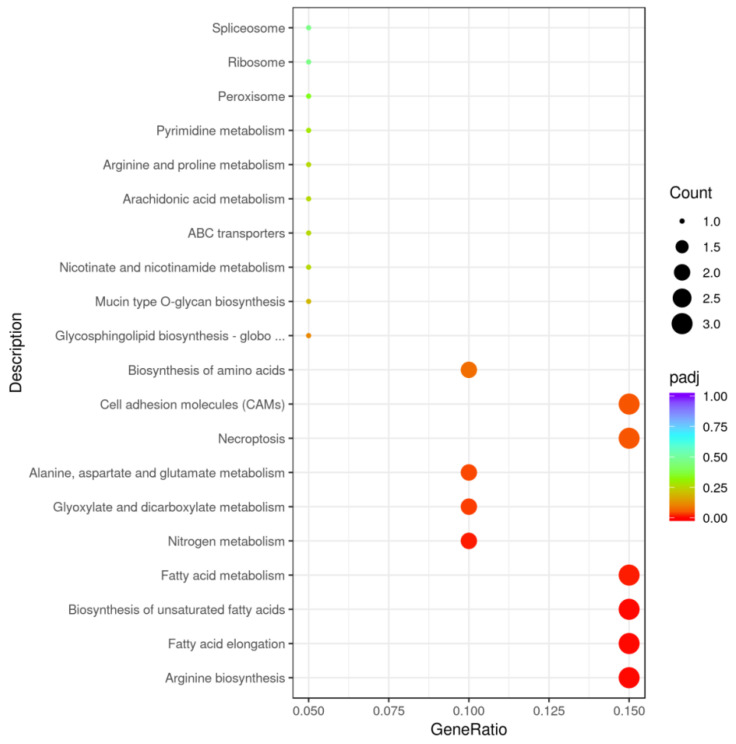
Expression trends of DEGs in RNA-seq and RT-qPCR in the CE groups (T vs. C).

To validate the RNA-seq results, ten DEGs were randomly selected for RT-qPCR analysis. The expression trends of all ten genes were consistent with those observed in the transcriptome data, confirming the reliability of the RNA-seq findings (Figure 5).

### 2.3. Proteome of Eyes Between Middle and Late Rotation CE

#### 2.3.1. Basic Proteome Data Analysisof Eyes with Middleand Late Rotation in CE

The six CE samples underwent quantitative analysis, revealing the following protein concentrations as measured by the BCA Protein Assay Kit: T1 = 4.18 μg/μL, T2 = 4.28 μg/μL, T3 = 4.71 μg/μL, C1 = 5.36 μg/μL, C2 = 4.50 μg/μL, and C3 = 3.64 μg/μL. All protein concentrations were above 3.6 μg/μL. The SDS-PAGE analysis of the samples showed clear protein bands, predominantly in the 15–25 kDa range, indicating intact proteins without degradation. These results met the quality control standards, confirming the samples were suitable for subsequent mass spectrometry analysis.

**Figure 6:**
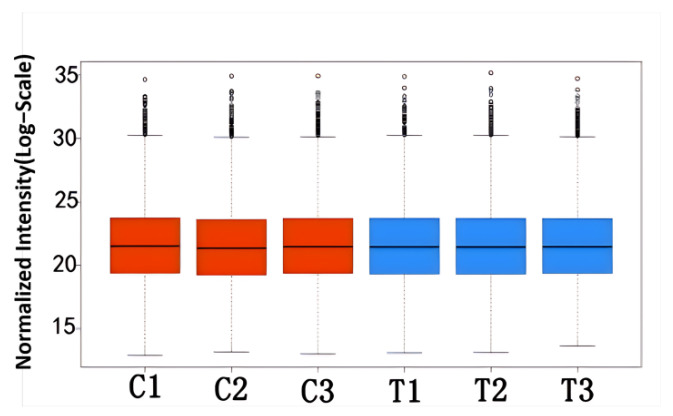
Boxplot of TMT proteomic data.

To explore the protein expression profiles associated with eye rotation in CEs, we conducted a proteomics study using TMT-labeled LC-MS/MS. The boxplot of the TMT protein quantification data (Figure 6) showed uniformity across the six samples, suggesting the data werereliable and consistent.

#### 2.3.2. DEPs in Different Eye Development Stages in CE

**Figure 7:**
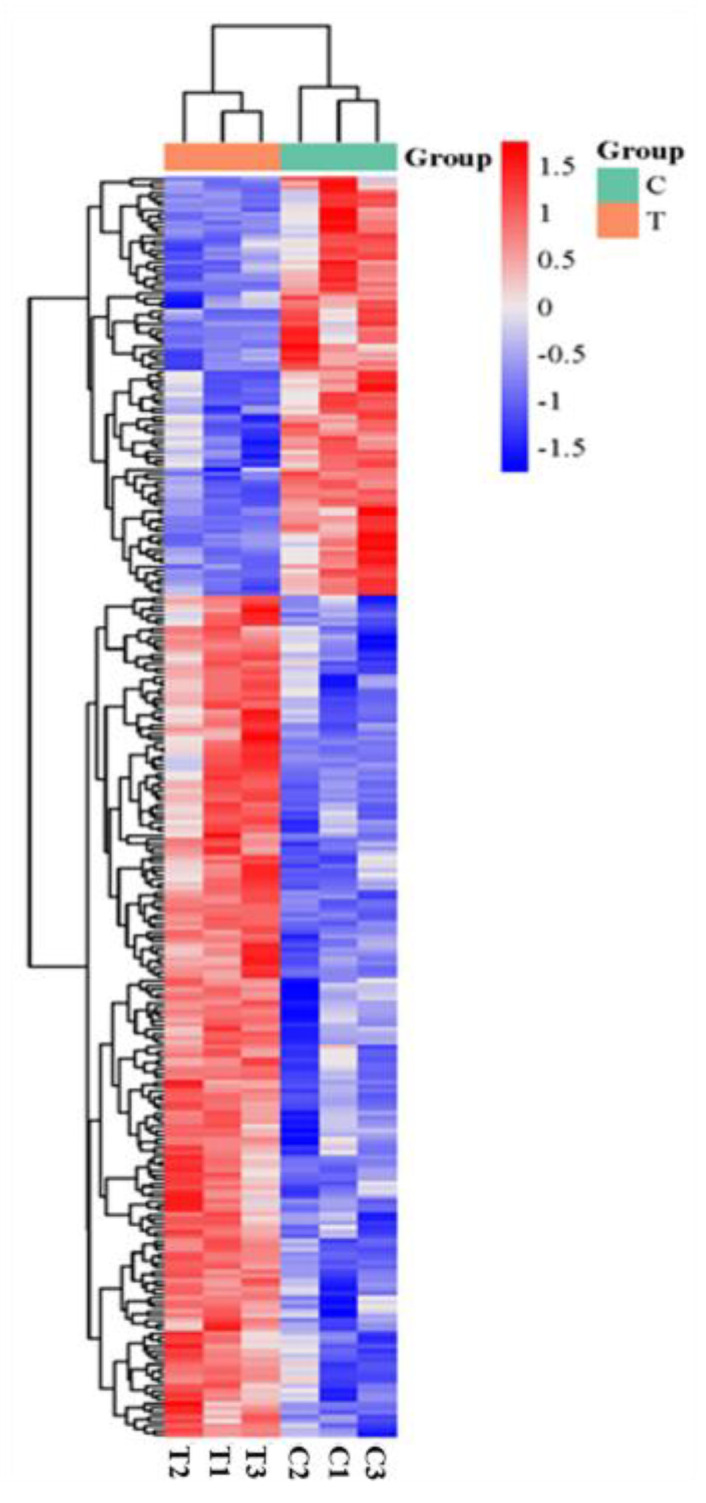
Heatmap and clustering analysis of DEPs.

**Figure 8:**
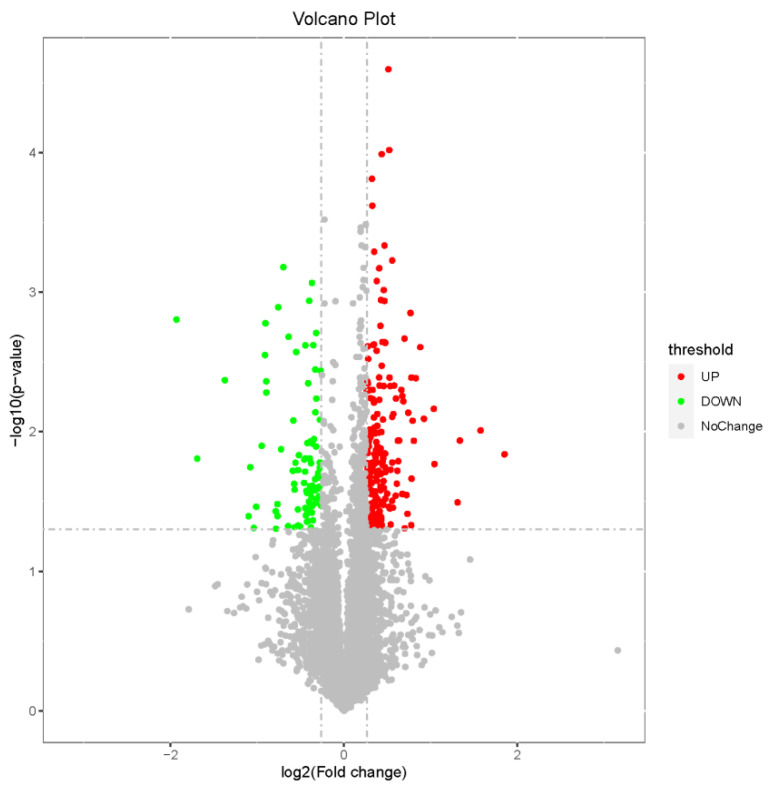
The volcano plot of DEPs identified with the eye rotating forward and upward in CEs. Note: The volcano plot of a total of 7717 proteins identified in T and C groups.The x-axis represents log2(Fold Change), and the y-axis represents −log10(*p*-value). The red dots indicate 191 significantly upregulated proteins (*p* < 0.05 and FC > 1.2), and the green dots indicate 95 significantly downregulated proteins (*p* < 0.05 and FC < 0.83). The gray dots represent proteins withnon-significant (*p* > 0.05 or 0.83 < FC < 1.2) differences in expression.

The TMT-based proteomics analysis of the six samples identified 38,566 unique peptides and 7717 proteins, representing 7678 unique genes that change during eye rotation. The proteomic data weresubmitted to the China National Center for Bioinformation database (OMIX007774). The clustering analysis of these DEPs showed that samples of the same type had similar expression patterns (Figure 7).The differential analysis of these data revealed 286 DEPs (191 upregulated and 95 downregulated in T groups compared with C groups) (Figure 8, Table S3).

#### 2.3.3. GO and KEGG Analysis ofDEPs of Eyes with Middleand Late Rotation in CE

**Figure 9:**
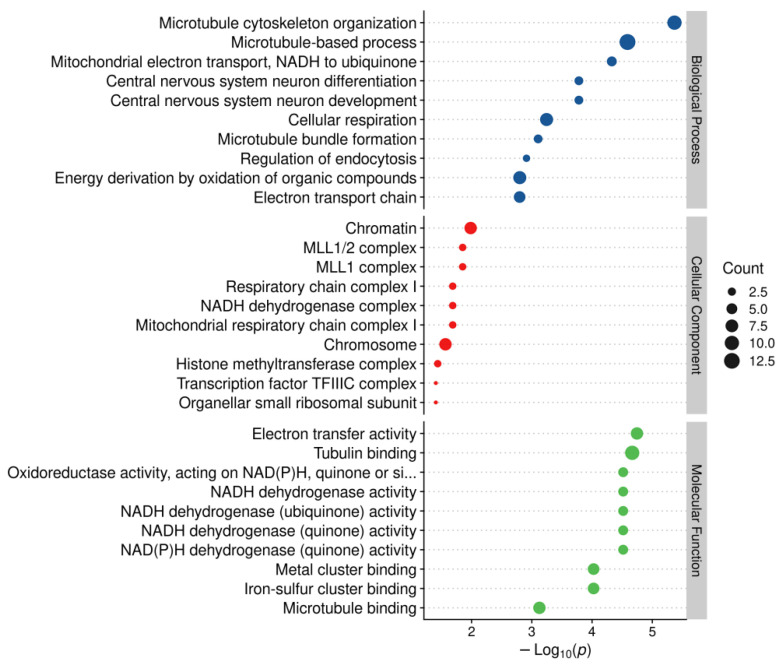
The top ten most significantly enriched GO terms for each category analysis with T vs. C. Note: The x-axis represents the enrichment ratio (−Log10*p*), and the y-axis represents the enrichment GO terms. The dot sizes represent the number of DEPs.

The 286 DEPs were significantly enriched (*p* < 0.05) in 107 Gene Ontology (GO) terms: 64 in biological process (BP), 28 in molecular function (MF), and 15 in cellular component (CC) categories (Table S3). The top 10 most significantly enriched GO terms for each category are shown in Figure 9.

In the biological process category, the most enriched terms included microtubule-based processes (13 proteins), microtubule cytoskeleton organization (ten proteins), central nervous system differentiation and development (nine proteins), and the regulation of endocytosis (two proteins).In the cellular component category, DEPs were predominantly associated with chromatin and chromosomes, with seven proteins each.In the molecular function category, the DEPs were mainly involved in tubulin binding (10proteins), microtubule binding (7proteins), and zinc ion binding (12 proteins).

**Figure 10:**
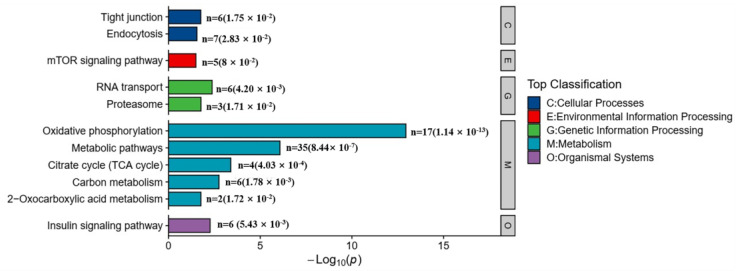
The top 11 most significantly enriched KEGG pathway analysis of DEPs with T vs. C. Note: The x-axis represents the enrichment ratio (−Log_10_(*p*)), and the y-axis represents the enrichment KEGG terms. The dot sizes represent the number of DEPs.

Additionally, the KEGG pathway analysis revealed significant enrichment (*p* < 0.05) in 11 pathways, primarily related to metabolism (64 proteins), signal transduction (6proteins), endocytosis (7proteins), and tight junctions (6proteins) (Figure 10). These DEPs were primarily associated with cytoskeleton structure, the central nervous system, and endocytosis.

#### 2.3.4. Protein Validation by PRM

To validate the TMT data, the expression levels of 17 eye-related proteins were quantified by LC-PRM/MS. Table 2 showed that the trends in protein expression for 14 proteins detected by PRM and TMT were largely consistent. However, there were three discrepancies between the TMT and PRM results, likely due to differences in the analytical and detection methods used. The Pearson correlation coefficient (R^2^ = 0.581) indicated a moderate positive correlation between the two data sets, confirming the reliability of these quantitative results.

### 2.4. Integrated Analysis of Protein and Transcriptional Data in Different Eye Development Stages in CE

**Figure 11:**
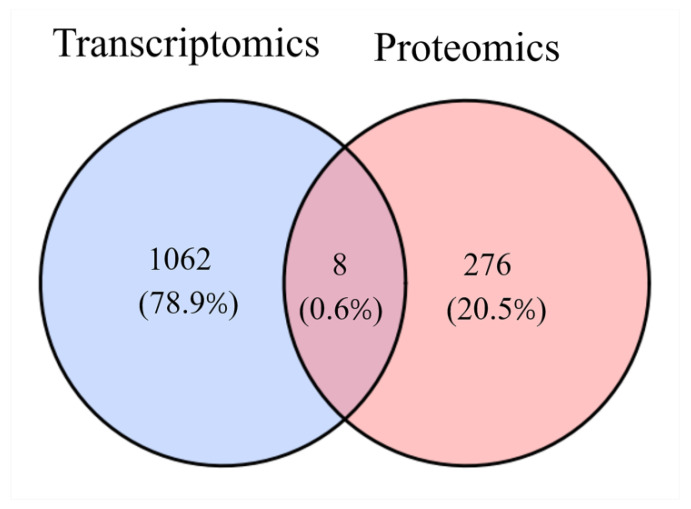
Venn diagram comparing DEGs/DEPs in the T vs. C transcriptome and proteome.

As no common genes were identified using the threshold (adjusted *p* < 0.05) for differentially expressed genes (DEGs), we applied a less stringent threshold (*p* < 0.05) to identify genes that were differentially expressed at the transcriptional level for comparison with protein expression data. Through this analysis, we found that 8genes exhibited differential expression at both the protein and transcriptional levels, 276 genes were differentially expressed at the protein level but not at the transcriptional level, and 1062 genes were differentially expressed at the transcriptional level but not at the protein level (Figure 11).

**Figure 12:**
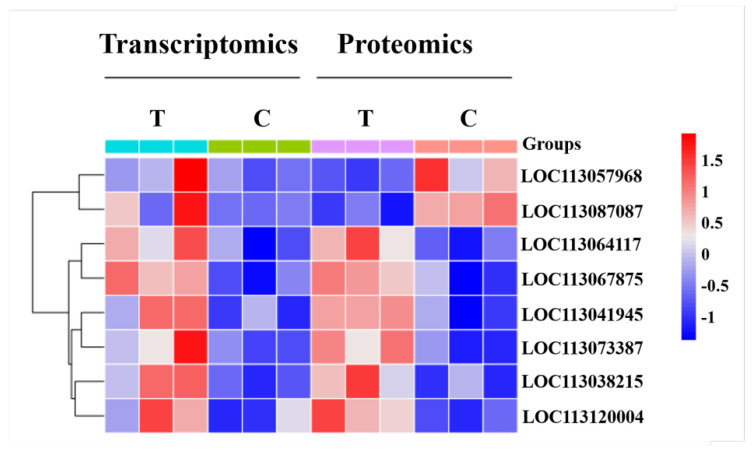
The cluster analysis of common significantly DEGs andDEPs between T and C groups in the combined with transcriptome and proteome data. Note: The x-axis represents the samples of T and C groups with research methods containing transcriptome and proteome, respectively, and the y-axis represents the clustering genes.

By integrating transcriptome and proteome data, we identified eight common DEGs. Among these, six genes showed consistent upregulation at both levels, while two genes, *LOC113057968* (*FHL1*) and *LOC113087087* (*MYDGF*), exhibited divergent expression patterns. A clustering analysis of these eight genes (Figure 12) revealed that *FHL1* and *MYDGF* clustered together, both of which were implicated in muscle [8] and cardiovascular diseases [9]. The remaining six genes formed a separate cluster. Notably, based on Gene Ontology (GO) annotation, *LOC113041945*, *FHL1*, *LOC113067875*, and *LOC113073387* were all associated with cytoskeletal functions or muscle development. *MYDGF* was reported to modulate adhesion responses in mice and inhibit apoptosis [10,11], while *Crabp2b* played a role in promoting optic nerve regeneration in goldfish [12].

**Figure 13:**
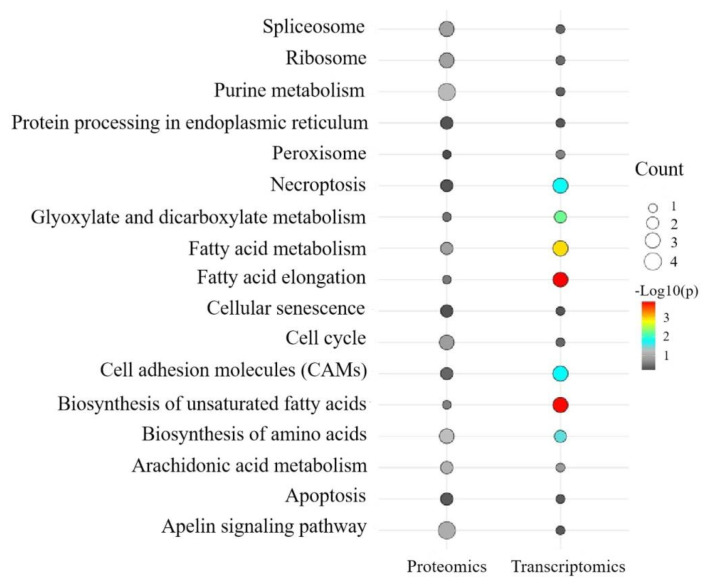
The bubble plot of common KEGG pathways between T and C groups in the combined with transcriptome and proteome data. Note: The color represents the enrichment ratio (−Log10*p*), and the dot sizes represent the number of DEGs or DEPs.

We further examined the common KEGG pathways by integrating the transcriptomic and proteomic data. The analysis revealed that apoptosis, necroptosis, and cell adhesion molecules were common pathways, although these pathways were not significantly enriched in the proteome data (Table S4, Figure 13).

## 3. Discussion

The CE goldfish is unique in that its eyes are permanently rotated to face upward. No matter from which angle you observe it, the eyes seem to be gazing directly at you. However, the molecular mechanisms behind the transition from forward-facing to upward-facing eyes remain poorly understood due to a lack of research in this area. Given the high genomic similarity among sibling individuals, phenotypic differences observed at various developmental stages are primarily driven by differential gene and protein expression. To investigate the underlying molecular mechanisms of this unique ocular trait, we performed both transcriptomic and tandem mass tag (TMT)-labeled proteomic analyses of the ocular tissues of CEs at different developmental stages. Here, we presented the first comprehensive transcriptomic and proteomic analysis comparing the ocular phases of anterior rotation and dorsal rotation in CEs.Itwas an exploratory study on the molecular mechanism underlying the formation of the goldfish’s celestial eyes.

The eye consisted of several key components: the cornea, iris, sclera, choroid, retina, optic nerve bundle, and vitreous body. The vitreous body, which accounted for approximately 80% of the eye’s volume, was a transparent, highly hydrated gel composed of 98% water along with collagen, hyaluronic acid, proteoglycans, and a small number of vitreous cells [13]. During CE development, we observed that the vitreous body gradually enlarged and protruded, while scleral proliferation was also apparent. As a result, the CE goldfish’s eye grew larger than that of a typical goldfish. Because the vitreous body contained few cells, the transcriptomic data primarily reflected the gene expression profiles associated with the ocular membranes and optic nerve bundle. The proteomic datareflected the whole eyeball’s protein changes in middle and later development stages of CEs.

### 3.1. Abnormalities in the Ocular Membrane Structure of CE in Retinal Degeneration, the Cornea and Sclera Undergoing Mechanical Stretching in the Development of CE

The comparative analysis of the transcriptomic and proteomic data identified 170 differentially expressed genes (DEGs), which were significantly enriched in GO terms and KEGG pathways, many of which were linked to structural abnormalities in the ocular membranes of the CE.

Previous studies reported retinal degeneration in CEs, specifically as the eyes rotated upward. This degeneration led to the destruction of photoreceptor cells and damage to the retinal pigment epithelium, which were then phagocytized by macrophages [4]. The analysis of our experiment was also consistent with it. The DEGs identified in our study were significantly enriched in GO terms related to cell adhesion, the cytoskeleton, and supramolecular polymers. Cell adhesion was critical for tissue formation and maintenance, particularly in the epidermis. Adhesion molecules were not merely “sticky” proteins—they regulated cytoskeletal elements and coordinate tissue development, structure, and physiology [14]. The cytoskeleton was a highly dynamic structure that not only provided structural scaffolding for cells but also participated in processes such as endocytosis, cell division, intracellular transport, adhesion, motility, force transmission, and adaptation to external stimuli. A robust connection between the intracellular cytoskeleton and the extracellular matrix was essential for maintaining the function of the retinal pigment epithelium and photoreceptors. Disruptions in these structures were frequently observed in retinal diseases [15]. In our analysis, four common genes (*LOC113041945*, *FHL1, LOC113067875*, and *LOC113073387*) involved in cytoskeletal development were significantly upregulated in both the transcriptomic and proteomic data, suggesting that the cornea and sclera in CEsmightalso undergo mechanical stretching.

In the supramolecular polymer category, six DEGs (*LOC113119645*, *LOC113062424*, *LOC113062422*, *LOC113110793*, *LOC113099666*, *LOC113094208*), all encoding keratin, were identified. Keratin played a vital role in the development and remodeling of the cornea [16]. Mutations in keratin genes had been linked to corneal diseases in humans [17]. Therefore, the three significantly enriched GO terms—cell adhesion, cytoskeleton, and supramolecular polymers—were strongly associated with the structural integrity of ocular membranes and likely contributed to the developmental and morphological changes observed in CEs.

Electron microscopy studies showed that the retina of CE goldfishwas thinner than that of a normal goldfish, with fewer photoreceptor cells. In some cases, retinal pigment epithelial cells and photoreceptor cells were replaced by macrophages [3]. Our findings revealed that many differentially expressed proteins (DEPs) were involved in endocytosis, suggesting an active role in phagocytosis. Additionally, DEGs enriched in necroptosis pathways indicated an increase in phagocyte production. Proteomic analysis identified nine proteins associated with central nervous system differentiation or development, suggesting a role in retinal photoreceptor degeneration.

Interestingly, in the five KEGG pathways related to nitrogen metabolism and synthesis, two genes (*LOC113063734* and *LOC113038790*) were identified as glutamine synthetase (GS). Research showed that decreased GS activity could lead to elevated intraocular pressure, increasing the risk of retinal damage [18]. Furthermore, GS expression was found to decrease as diabetic retinopathy progresses [19], with mouse models demonstrating a decline in GS activity and transcriptional expression as the disease worsens [20]. In our study, both *LOC113063734* and *LOC113038790* (GS) were expressed at higher levels in the T group compared to the C group. This suggested that in the later stages of eye rotation in CEs, retinal degeneration might be more severe than in the earlier stages.

### 3.2. Arginine BiosynthesisMight Be Closely Linked to the Development of CE

The KEGG pathway enrichment analysis revealed that amino acid synthesis and metabolism were the primary pathways involving DEGs in CEs. Both CE and telescope goldfish (TE),which also exhibited protruding eyes, shared similar abnormal pathways [21], with DEGs in both species enriched in amino acid metabolism. Specifically, in the KEGG pathway enrichment analysis of the BSA mapping candidate region for the telescope eye, the most significantly enriched pathway was arginine biosynthesis, followed by alanine, aspartate, and glutamate metabolism [21]. In our study, arginine biosynthesis also ranked first; nitrogen metabolism ranked fourth; alanine, aspartate, and glutamate metabolism ranked seventh; and amino acid biosynthesis ranked tenth, involving only three genes: *LOC113063734* (GS-like), *LOC113038790* (GS-like), and *LOC113078755* (NOS2a, nitric oxide synthase 2a, inducible).

Arginine biosynthesis, nitric oxide (NO), and GS were all critical to the development and function of ocular tissues. Arginine metabolism was linked to diabetic retinopathy [22], and it played an important role in collagen secretion by corneal fibroblasts [23] and maintaining immune privilege in the cornea [24]. Inducible nitric oxide synthase (NOS2) was associated with glaucoma [25] and was involved in regulating retinal blood flow [26]. NO had an antiproliferative effect on retinal pigment epithelial cells [27] and contributed to the regulation of aqueous humor outflow [28]. GS was also implicated in retinal diseases [19].

Previous studies suggested that myopia resulted from increased vitreous fluid, leading to ocular enlargement as well as from the thin, fragile cornea and sclera associated with vitreous expansion in myopic chicks and CEs [5,29]. Our findings, combined with the transcriptomic analysis, showed significant differences in protein synthesis and metabolism between the T and C groups of CEs, particularly in the arginine biosynthesis pathway. We hypothesized that abnormalities in arginine synthesis might be closely linked to the progressive increase in vitreous fluid, leading to the enlargement of the vitreous body and the proliferation of ocular membranes, such as the retina and cornea. During this process, altered amino acid synthesis and metabolism pathways likely played a key role in the development and rotation of CEs.

### 3.3. Comparison of CE and Telescope Goldfish (TE)—lrp2 Gene and Abnormal Lipid Metabolism in the Development of CE and TE

Both CE and TE goldfish exhibitedabnormal functional issues in their eyes. In CE goldfish, retinal degeneration occurred at varying stages of development, leading to narrowed vision and reduced light sensitivity [2]. In contrast, TE goldfish also had protruding eyes, and no retinal degeneration was observed [4]. However, the TE was characterized by severe myopia [30]. Despite both conditions involving protruding eyes, their morphological developments differ: in CEs, the eyes gradually rotated upward until they aligned parallel to the back, maintaining this upward orientation. In contrast, the TE did not show this characteristic. Additionally, these two types of abnormalities emerged at different points in history, suggesting that they were distinct strains [31].

Previous studies identified mutations linked to TEs. For instance, Kon et al. [32] reported that a mutation in the 45th intron of the *lrp2aL* gene in black TEs was associated with a 13-kb retrotransposon insertion, which led to a premature termination of the gene’s coding sequence. Additionally, a study by Yu et al. [21] identified a single base mutation in the 73rd exon of the *lrp2aB* gene in TEs, resulting in a stop codon that contributed to the TE phenotype. However, no reports currently have existed on the causal mutations in CEs. Notably, Kon et al. [32] mentioned that the protein encoded by the *lrp2aL* gene in albino CEs was shorter than that in black TEs. Therefore, the *lrp2* gene, primarily involved in lipid metabolism, also might play a role in regulating the development of CEs.

The transcriptomic analysis revealed that the *LOC113108271* (*lrp2*)gene showed no significant difference between the T and C groups (TvsC_*p* = 0.616), and corresponding protein sequences were not identified in the proteomic analysis. This suggested that the *lrp2* gene was expressed similarly in both developmental forms of CEs without significant variation and that the corresponding protein sequences were not detected in either eye form. This finding contradicted the conclusions of Kon et al. [32], who reported that the *lrp2* gene *encoded* a shorter protein sequence in albinismus CEs, which might be attributed to strain or developmental stage differences.In other words, the *lrp2* gene in the line of CEswe used in this experiment did not encode the corresponding protein sequence. Therefore, this gene could be considered as a key candidate gene for the formation of CEs for subsequent research and validation.

The KEGG pathway analysis of transcriptomic data revealed three pathways significantly related to lipid metabolism: fatty acid elongation, the biosynthesis of unsaturated fatty acids, and fatty acid metabolism. Three differentially expressed genes (DEGs) were identified in these pathways: *LOC113054720* (elongation of very long chain fatty acids protein 7-like), *LOC113110194* (elongation of very long chain fatty acids protein 7-like), and *LOC113112742* (elongation of very long chain fatty acids protein 6-like). In TEs [33], lipid metabolism pathways were also significantly enriched, indicating that abnormal lipid metabolism was closely associated with the development of enlarged and protruding eyes in both CEs and TEs. Fatty acids were essential for maintaining retinal integrity [34], as they were abundant in both normal and pathological retinas, primarily consisting of polyunsaturated fatty acids [35]. Comparisons between the T and C groups further revealed differences in unsaturated fatty acid synthesis pathways. Increasing evidence suggested that abnormal lipid metabolism was linked to retinal physiology and the pathology of retinal vascular diseases [36]. Abnormal lipid accumulation in the eye damaged photoreceptor cells, impairing light transmission and potentially leading to myopia or blindness [37]. In mouse eye models, increased phagosome accumulation was accompanied by reduced fatty acid oxidation and lipid deposition in the retinal pigment epithelium.

When comparing the two developmental forms of CEs, the three lipid metabolism-related genes were downregulated in the C group, suggesting a slowdown in lipid synthesis. This could imply that during the mid-developmental stage (T group), the retina was actively proliferating to repair degeneration-induced damage, while later stages exhibited a reduction in lipid metabolism due to phagosome accumulation and lipid buildup.

### 3.4. Energy Metabolism WasDownregulated in the Later Stages of CEDevelopment Compared to the Mid-Development Stages

The KEGG enrichment analysis ofDEPsidentified four pathways significantly related to energy metabolism (Figure 10). The GOanalysis revealed that two of the top ten biological processes (BPs) were significantly associated with energy metabolism (Figure 9). Additionally, two cellular components (CCs) related to energy metabolism were identified (Figure 9). Five molecular functions (MFs) also showed significant enrichment(Figure 9). The KEGG enrichment analysis of DEGs further revealed significant pathways such as arginine biosynthesis;fatty acid metabolism;nitrogen metabolism;glyoxylate and dicarboxylate metabolism; and alanine, aspartate, and glutamate metabolism—all of which were involved in energy metabolism. Overall, these results indicated that energy metabolism was downregulated in the C groups compared to the T groups.

Arginine served as a precursor to creatine, which was converted to phosphocreatine, a key energy storage molecule found in skeletal muscle and the central nervous system [38]. Alanine, aspartate, and glutamate were glucogenic amino acids that were converted into oxaloacetate, α-ketoglutarate, or succinyl CoA, which then were oxidized in the TCAcycle and oxidative phosphorylation to produce ATP. Studies of diabetic retinopathy (DR) suggested that elevated levels of these amino acids were associated with increased NADH levels and a higher NADH/NAD ratio, which reduced TCA cycle activity and downregulates catabolism in DR patients. Moreover, endothelial dysfunction in DR was accompanied by decreased amino acid catabolism and reduced antioxidant synthesis, including threonine and glutathione [39]. Thus, retinal endothelial dysfunction in the C group appeared more severe than in the T group, contributing to a decline in energy metabolism.

## 4. Materials and Methods

### 4.1. CE Culturing and Sampling

The experimental fish were sourced from the Ornamental Fish Research Laboratory at the Beijing Academy of Agricultural and Forestry Sciences, where the breeding experiment was conducted. The goldfish were maintained in glass aquariums (1.2 m × 0.6 m × 0.45 m) indoors, with a water depth of 0.35 m. The water temperature fluctuated naturally with the changing seasons, and the aquariums were exposed to natural light. The water used for the aquariums was aerated underground water, which was left to stand for over 48 h prior to use. During the rearing period, the fish were fed according to their health condition, ensuring proper nutrition.

Water quality was regularly monitored and maintained within the following ranges: pH 7.0–8.4, dissolved oxygen 7.70–9.70 mg/L, nitrite concentration < 0.02 mg/L, and ammonia nitrogen concentration < 0.15 mg/L.

During the breeding season, a pair of healthy CE goldfish was selected for artificial insemination. Following natural incubation, 84 juvenile fish were randomly selected and acclimated in new aquariums. Throughout the breeding experiment, phenotypic observations of the developmental processes, specifically eye rotation, were conducted on the CE.

**Figure 14:**
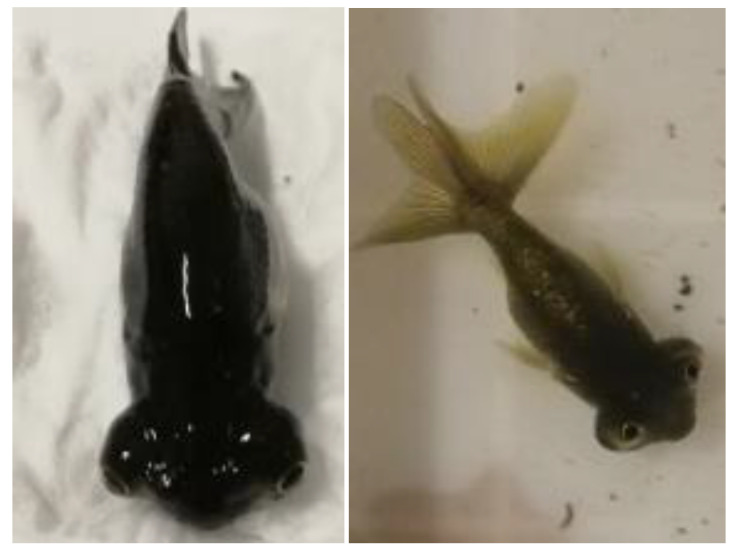
The mid and late stages of eye development in celestial-eye goldfish. Note: (**Left**): CE goldfish with forward eyes (T group), (**Right**): CE goldfish with upward eyes (C group).

At 110 days post-hatching (dph), three goldfish exhibiting forward-facing eyes and three exhibiting upward-facing eyes (Figure 14) were randomly selected for sampling. These fish were then divided into two groups: group T (T1, T2, T3) and group C (C1, C2, C3). After euthanizing the fish with MS-222 anesthesia, their eye tissues were promptly collected and flash-frozen in liquid nitrogen. The eye samples were then ground under liquid nitrogen, and each sample was split into two portions: one for transcriptomic high-throughput sequencing and the other for tandem mass tag (TMT)-based quantitative proteomic analysis.

### 4.2. RNA Extraction, Transcriptome Sequencing, and Bioinformatic Analysis of Celestial Eye in the Mid- and Late-Development Stages

The reagents, instruments, methods, and procedures used for RNA extraction, transcriptome sequencing, and bioinformatic analysis of the CE goldfish eye tissue samples were identical to those described for fish skin by Rongni Li et al. [40]. DEGs, key biochemical and metabolic pathways, and signaling pathways involved in eye development were identified.

### 4.3. Verification of RNA-Seq

To eliminate residual genomic DNA, each RNA sample was treated with the PrimeScript™ RT Reagent Kit with gDNA Eraser (Takara, Japan) and subsequently reverse transcribed into complementary DNA (cDNA). A total of 10 genes were randomly selected for verification, and RT-qPCR primers were designed using Primer Express^®^ Software Primer 5.0 (Table S1). RT-qPCR was conducted using a Stratagene Mx3000P instrument (Agilent, Santa Clara, CA, USA). The relative expression of each gene was normalized to the internal control gene, *β-actin*, using the comparative Ct method. Each experiment was performed with three biological replicates.

### 4.4. Tandem Mass Tag (TMT)-Based QuantitativeProteomic Analysis of Celestial Eye in the Mid- and Late-Development Stages

The CE goldfish eye samples (three biological replicates per group) were homogenized in SDT buffer via sonication on ice, followed by centrifugation. Protein concentration in the supernatant was determined using the BCA method. To each sample (300 μg of total protein), 1M DTT was added to achieve a final concentration of 100 mM, and the samples were then heated. After multiple rounds of ultrafiltration (Microcon units, 10 kD) with UA buffer, IAA was added to the retentate to a final concentration of 50 mM, and the ultrafiltration tubes were kept in the dark for 30 min. The retentate was then washed with UA buffer and NH_4_HCO_3_ buffer, followed by centrifugation. Following digestion with 6 μg of trypsin, peptides were collected, acidified with 0.1% TFA, desalted using a C18 Cartridge, vacuumfreezedried, and reconstituted in TFA buffer. The final peptides were further desalted using a desalting spin column and quantified.

Each 100 μg peptide mixture was labeled with TMT reagents according to the manufacturer’s instructions (Thermo Fisher Scientific, Waltham, MA, USA). Peptides were fractionated using the Pierce™ High pH Reversed-Phase Peptide Fractionation Kit (Thermo Fisher Scientific), followed by LC-MS/MS analysis on a Q Exactive HF-X mass spectrometer coupled withan Easy nLC system. Mobile phases were prepared as follows: phase A, 99.9% water with 0.1% formic acid; phase B, 95% acetonitrile, 0.1% formic acid, and 4.9% water. Samples were loaded onto a C18 Trap column (100 μm × 20 mm, 5 μm) and separated on a C18 analytical column (75 μm × 150 mm, 3 μm) using a gradient elution method. The mass spectrometry acquisition parameters were set as follows: positive mode, full scan range from *m*/*z* 300 to 1800 with a resolution of 60,000 (at *m*/*z* 200), AGC target = 3 × 10^6^, and maximum ion injection time = 50 ms. The top 20 most intense parent ions were selected for HCD fragmentation, and the data were acquired at a resolution of 15,000 (at *m*/*z* 200), with an AGC target of 1e5 and a maximum ion injection time of 50 ms. The normalized collision energy for HCD fragmentation was set to 32%.

Raw LC-MS/MS data files were imported into Proteome Discoverer software (version 2.4, Thermo Scientific) and searched using the Sequest HT search engine against the Uniprot-*Carassius auratus* (Goldfish) database [7957]-84849-210615.

### 4.5. Verification of TMT Proteomic Data by Parallel Reaction Monitoring (PRM)

To validate the proteins identified by TMT quantification, 17 proteins were selected for further quantitative analysis using LC-PRM/MS, based on the methodology described by Li et al. [41], with minor modifications. Briefly, 2 µg of peptide samples were analyzed via LC-PRM/MS. Chromatographic separation was conducted on a nano-flow Easy nLC 1200 Chromatography System (Thermo Scientific) with the following buffer solutions: Buffer A, 0.1% formic acid in water; Buffer B, 0.1% formic acid, acetonitrile, and water (95% acetonitrile). The chromatography column was equilibrated with 95% Buffer A. After sample loading, peptides were separated using a C18 analytical column (75 μm × 150 mm, 3 μm, Dr. Maisch GmbH, Ammerbuch-Entringen, Germany) at a flow rate of 300 nL/min. The gradient was as follows: 0–5 min, 2–5% Buffer B; 5–45 min, 5–23% Buffer B; 45–50 min, 23–40% Buffer B; 50–52 min, 40–100% Buffer B; and 52–60 min, 100% Buffer B. PRM analysis was performed using a QExactive HF-X mass spectrometer (Thermo Scientific). The full scan range was 300–1200 *m*/*z*, with a resolution of 60,000 at *m*/*z* 200, AGC target = 3 × 10^6^, and maximum ion injection time = 50 ms. The top precursor ions were selected for MS2 analysis, with a resolution of 30,000 at *m*/*z* 200, AGC target = 1 × 10^6^, and maximum ion injection time = 100 ms. HCD was used for fragmentation, with a normalized collision energy of 27. Data were processed for quantification using Skyline 4.1 [42].

### 4.6. Statistical Analysis

For transcriptomic analysis, differential expression of genes between groups (three biological replicates per group) was determined using the DESeq2 R package (version 1.16.1). The Benjamini–Hochberg procedure was applied to adjust the *p*-values, controlling the false discovery rate (FDR). Genes with adjusted *p* < 0.05 and fold change (FC) > 2 were considered differentially expressed (DEGs).

For proteomic analysis, peptide-spectrum matches (PSMs) and proteins were identified with an FDR < 1%. Protein quantification was statistically analyzed using a *t*-test, with *p* < 0.05 and FC > 1.2 as the threshold for differential protein expression (DEPs).

To assess the biological significance of the DEGs and DEPs in the context of celestial-eye formation, GOenrichment analysis and Kyoto Encyclopedia of KEGG pathway analysis were performed using the ClusterProfiler R package (version 3.4.4). For DEGs, the thresholds for significant GO and KEGG enrichment were set to adjusted *p* < 0.05. For proteomic dat, GO and KEGG enrichment analyses were performed based on Fisher’s exact test, using all identified proteins as the background dataset, with a *p* < 0.05 considered significant.

### 4.7. Integrated Analysis of DEGs and DEPs and TMT and RNA-Seq Data

After applying the screening criteria for DEGs and DEPs (as described in Section 4.7), no significant overlap was found between the differentially expressed genes from TMT and RNA-seq data. Additionally, KEGG pathway analysis of the two omics datasets showed no common significantly enriched pathways. Therefore, the DEGs screening criteria were adjusted to *p* < 0.05 and FC > 2, and common differential genes were re-assessed across both datasets.

## Figures and Tables

**Table 1:** Data statistics of transcriptome sequencing of eye samples from CE.

Sample	Raw_Reads	Clean_Reads	Q20	Q30	GC_Pct	Total_Map	Exon
C1	43,690,134	42,323,868	97.92	93.94	47.24	91.88%	89.19%
C2	45,945,686	44,625,592	97.71	93.48	44.28	89.66%	85.05%
C3	44,798,590	43,225,138	98.09	94.4	48.04	92.88%	90.54%
T1	45,146,806	43,811,750	97.73	93.73	47.34	92.56%	89.44%
T2	43,529,570	42,244,548	98.02	94.17	47.49	92.38%	90.87%
T3	42,266,654	40,614,504	97.85	93.82	45.82	90.53%	87.98%

**Table 2:** Comparison of quantification results between TMT and PRM analyses for 17 selected proteins.

Accession	Protein Names	Gene Names	PRM ^a^	TMT ^b^
A0A6P6IU08	15-hydroxyprostaglandin dehydrogenase [NAD(+)]-like	*LOC113038215*	1.04	1.30
A0A6P6IY34	Isocitrate dehydrogenase [NAD] subunit, mitochondrial	*LOC113038769*	0.96	1.21
A0A6P6JK81	Clathrin light chain	*LOC113044322*	1.33	1.37
A0A6P6JQ89	Succinate dehydrogenase [ubiquinone] iron-sulfur subunit, mitochondrial	*LOC113045612*	0.71	0.77
A0A6P6KEK8	Carnitine O-palmitoyltransferase	*LOC113051329*	0.92	0.70
A0A6P6LA17	four and a half LIM domains protein 1-like isoform X2	*LOC113057968*	0.36	0.77
A0A6P6LCR7	alpha-crystallin A chain-like	*LOC113058390*	0.88	1.31
A0A6P6MA34	brefeldin A-inhibited guanine nucleotide-exchange protein 1-like isoform X2	*LOC113066031*	0.42	1.31
A0A6P6MCP1	Catalase	*LOC113066695*	1.08	1.27
A0A6P6MEJ7	Small monomeric GTPase	*LOC113067076*	0.95	0.80
A0A6P6N3I1	Arrestin-C	*LOC113074409*	1.20	1.34
A0A6P6N7D3	Succinate dehydrogenase [ubiquinone] iron-sulfur subunit, mitochondrial	*LOC113076629*	0.55	0.81
A0A6P6PVN9	Voltage-dependent anion-selective channel protein 3	*LOC113106967*	1.06	1.44
A0A6P6Q4R0	myc box-dependent-interacting protein 1-like isoform X2	*LOC113108888*	1.12	1.26
A0A6P6RIT1	2-phospho-D-glycerate hydro-lyase	*LOC113119875*	1.22	1.23
A0A6P6RK04	cellular retinoic acid-binding protein 2-like	*LOC113120004*	1.84	1.37
A0A6P6RKE7	ras-related protein Rab-5A	*LOC113120091*	1.27	1.55

^a^ Fold changes in protein abundance from the comparisons between T and C groups, analyzed using tandem mass tag (TMT) proteomics. ^b^ Fold changes in protein abundance from the comparisons between T and C groups, determined using parallel reaction monitoring (PRM).

## Data Availability

The authors confirm that the data supporting the findings of this study are available within the article. The raw sequence data of transcriptome and proteome reported in this paper have been deposited in the Genome Sequence Archive (Genomics, Proteomics & Bioinformatics 2021) in National Genomics Data Center (Nucleic Acids Res 2022), China National Center for Bioinformation/Beijing Institute of Genomics, Chinese Academy of Sciences (GSA: CRA020175 and OMIX007774), that ispublicly accessible at https://ngdc.cncb.ac.cn/gsa.

## Supplementary Materials

The following supporting materials were uploaded at: https://www.mdpi.com/article/10.3390/ijms26020466/s1.
